# Anti‐*Toxoplasma gondii* activity of rose hip oil–solid lipid nanoparticles

**DOI:** 10.1002/fsn3.4043

**Published:** 2024-02-20

**Authors:** Hanieh Mohammad Rahimi, Zahra Hesari, Elnaz Sadat Mirsamadi, Sara Nemati, Hamed Mirjalali

**Affiliations:** ^1^ Foodborne and Waterborne Diseases Research Center Research Institute for Gastroenterology and Liver Diseases, Shahid Beheshti University of Medical Sciences Tehran Iran; ^2^ Department of Pharmaceutics School of Pharmacy, Guilan University of Medical Sciences Rasht Iran; ^3^ Department of Microbiology, Faculty of Medicine Tehran Medical Sciences, Islamic Azad University Tehran Iran

**Keywords:** herbal medicine, nanomedicine, rose hip oil, solid lipid nanoparticles, toxoplasmosis

## Abstract

*Toxoplasma gondii* is a highly prevalent pathogen, reported from almost all geographical regions of the world. Current anti‐*T. gondii* drugs are not effective enough in immunocompromised patients, encephalitis, chorioretinitis, and congenital toxoplasmosis. Therefore, the prescription of these drugs has been limited. Rose hip oil (RhO) is a natural plant compound, which shows antibacterial, anticancer, and anti‐inflammatory activities. In the current study, the anti‐*T. gondii* and cell toxicity effects of solid lipid nanoparticles (SLNs) loaded by RhO (RhO‐SLNs) were evaluated. Emulsification sonicated‐homogenization method was used to prepare SLNs. RhO‐SLNs were characterized, and their anti‐*T. gondii* and cell toxicity effects were evaluated using in vitro analyses. The particle size and the zeta potential of the nanoparticles were 152.09 nm and −15.3 mV nm, respectively. The entrapment efficiency percentage was 79.1%. In the present study, the inhibitory concentration (IC)_50_ against *T. gondii* was >1 μg/mL (*p*‐value <.0001). The cell toxicity assay showed cytotoxicity concentration (CC)_50_ >10 mg/mL (*p*‐value = .017). In addition, at least 75% of *T. gondii*‐infected Vero cells remained alive at concentrations >10 mg/mL. The concentration of 1 mg/mL showed highest anti‐*Toxoplasma* activity and lowest cell toxicity against the Vero cell. Our findings suggest that carrying natural plant compounds with SLNs could be considered an effective option for treatment strategies against *T. gondii* infections.

## Graphical abstract

Natural products seem to be alternatives for chemical agents in the pharmaceutical industry. Solid lipid nanoparticles (SLNs) reduce the degradation of therapeutic agents and increase the clearance rate of drugs. RhO‐SLNs showed high *T. gondii* effects with low cell toxicity.

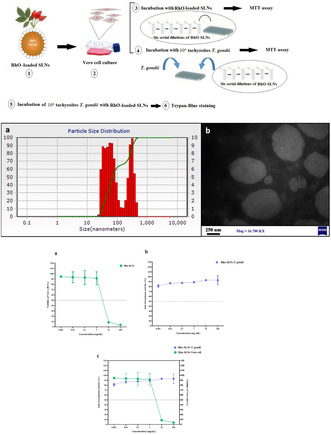

## 1 INTRODUCTION

Toxoplasmosis is a cosmopolitan parasitic disease caused by *Toxoplasma gondii*, which has almost been described in all warm‐blooded animals (de Barros et al., 2022). The main clinical manifestations range from mild influenza‐like symptoms to severe complications, particularly in subjects with comprised immune system (Joynson, 2001). The principal transmission routes of *T. gondii* are congenital, eating uncooked foods, accidental ingestion of sporulated oocysts via contaminated soil and unwashed fruits and vegetables, and blood transfusion (Al‐Malki, 2021; Pinto‐Ferreira et al., 2019).

The treatment of toxoplasmosis is necessary in symptomatic, immunocompromised patients, and children younger than 5 years (Konstantinovic et al., 2019). Pyrimethamine is the most effective drug against toxoplasmosis (Dunay et al., 2018). The current gold standard for treating toxoplasmosis is a combination of pyrimethamine and sulfadiazine (pyr‐sulf), which targets the tachyzoite stage (Konstantinovic et al., 2019). However, high treatment failure rate against chronic infection of this combination has been reported (Ben‐Harari et al., 2017). Moreover, some severe side effects such as hepatic necrosis, thrombocytopenia, and teratogenic potential have been reported (Dunay et al., 2018; Katlama et al., 1996). Additionally, different severe adverse effects have been reported from azithromycin, clarithromycin, and trimethoprim–sulfamethoxazole (Montazeri et al., 2017; Wei et al., 2015). Although several drugs are commercially available, undesirable side effects have increased research to develop novel therapeutic agents for effective treatment of encephalitis, chorioretinitis, and congenital toxoplasmosis (Aspinall et al., 2002; Ben‐Harari et al., 2017; Béraud et al., 2009; Doliwa, Escotte‐Binet, et al., 2013; Doliwa, Xia, et al., 2013; McFadden et al., 2000; Meneceur et al., 2008; Shammaa et al., 2021; Torre et al., 1998).

Medicinal plants are the primary source of medicine, which have traditionally been employed for the treatment of human diseases for thousand years, all over the world. These natural products have been proved to be an alternative for chemical agents in the pharmaceutical industry (Molaei et al., 2021; Petrovska, 2012; Sofowora et al., 2013). Recently, natural extracts of plants have been considered as anti‐*Toxoplasma* components (Choi et al., 2013; Kavitha et al., 2012; Khamesipour et al., 2022; Mohammad Rahimi et al., 2020; Nemati et al., 2022). *Rosa rubiginosa* (*Rosa eglanteria* L.), known as rose hips (*Rosa* spp., *Rosaceae*), are wild rose bushes with more than 100 species, which are widely used in the preparation of cosmetic products and the food industry (Franco, Pinelo, et al., 2007; Loetscher et al., 2013; Santana et al., 2016). Rose hip fruits have been very popular in different countries due to their antioxidant properties and having various minerals, high amount of vitamin C, ascorbic acid, carotenoids, and phenols (Demir et al., 2014; Ercisli, 2007; Franco, Pinelo, et al., 2007; Franco, Sineiro, et al., 2007; Roman et al., 2013). Many in vivo and in vitro studies have been conducted on the different compounds of rose hip oil (RhO) extract (Mármol et al., 2017; Yi et al., 2007). A significant effect of RhO extract was described in the treatment of lesions such as eczema and trophic skin injuries (Lei et al., 2019). Moreover, antibacterial (Rovnã et al., 2015), anticancer (Tumbas et al., 2012), antioxidant (Abdelbaky et al., 2021), and anti‐inflammatory (Yan et al., 2013) properties of rose hip oil extracts have been evaluated. However, there is no evidence indicating antiparasitic effects of rose hip extracts.

In recent years, the development of nanotechnology sciences has resulted in the designing of novel drug delivery carriers, leading to development of pharmaceutical and cosmetic‐sanitary products (Cai & Chen, 2007; Zhang et al., 2008). Liposomes, microemulsions, multiple emulsions, and solid particles are the most popular drug delivery carriers. In the early 1990s, solid lipid nanoparticles (SLNs), as a colloidal carrier with a particle size of 50–1000 nm, were designed and manufactured (Müller et al., 2002). SLNs are prepared from physiological lipids and dispersed well in aqueous surfactant solution (Musielak et al., 2022). The advantages of SLNs are small size, high surface‐to‐volume ratio, phase interaction at the particle level, high drug carrying capacity, appropriate release profile, ability to be used in the field of targeted drug delivery, high physical stability, and high biocompatibility (Ezzati Nazhad Dolatabadi et al., 2015; Uner & Yener, 2007). In addition, SLNs reduce the toxicity and degradation of a therapeutic agent, while increasing the clearance rate of the drug (Ghasemiyeh & Mohammadi‐Samani, 2018).

Although antibacterial effects of RhO have been evaluated, there is no evidence indicating the application of this extract on parasites like *Toxoplasma*. In addition, employing lipid nanocarriers like SLNs can increase efficiency and decrease cell toxicity of herbal extracts. Therefore, employing a new formulation based on SLNs for target delivery of RhO seems to be novel in the drug delivery area. In this study, we evaluated the effect of RhO‐SLNs against *T. gondii* RH strain and Vero cell line.

## 2 MATERIALS AND METHODS

### 2.1 Preparation of RhO‐SLNs

For the preparation of SLNs loaded RhO (RhO‐SLNs), an emulsification sonicated homogenization method was used (Nemati et al., 2022; Trotta et al., 2005). For this purpose, 10‐mL methanol (Merck, Darmstadt, Germany) was used to dissolve 1‐mL RhO (Lavandor Co. Tehran, Iran). RhO‐SLN preparation was performed based on the protocols mentioned elsewhere (Nemati et al., 2022).

### 2.2 Physical characterization of RhO‐SLNs

#### 2.2.1 Particle size, polydispersity index (PDI) and zeta potential

A Zetasizer 1,033,439 (Malvern Instrument, UK) was utilized to determine the particle size and zeta potential of nanoparticles. In this regard, 20 μL of the sample was suspended in 1 mL of double‐distilled water and the average particle size was calculated as mentioned previously (Nemattalab et al., 2022).

#### 2.2.2 Transmission electron microscopy (TEM) and entrapment efficiency (EE)

Upon uranyl acetate staining and former resin coating, the shape and morphology of nanoparticles were characterized using a TEM microscope (Zeiss‐EM10C‐100 KV, Germany). To calculate the EE percentage, 1 g of RhO‐SLN was dispersed in 10 mL of distilled water (containing 2% v/v tween 80), centrifuged at 6000 rpm for 20 min at 25°C, and the not encapsulated RhO and the entrapped oil percentage was calculated as mentioned previously (Nemattalab et al., 2022; Pinheiro et al., 2020).

#### 2.2.3 Release kinetics

To evaluate the release kinetics of the drug, 1.5 g of RhO‐SLNs was packed into a dialysis bag with a molecular weight cutoff of 14 kDa (Sigma, Steinheim, Germany). The bag was immersed in receptor medium (100‐mL distilled water containing 2% v/v tween 80), and the analyses of the RhO content were performed at different time points (Nemattalab et al., 2022; Wakade & Shende, 2021), based on available mathematical kinetic models (Mortazavi & Mortazavi, 2020).

### 2.3 Biology laboratory experiments

#### 2.3.1 Parasites

Tachyzoites of *T. gondii* (RH strain) were washed with sterile PBS (pH: 7.4) and counted. In this study, the kidney fibroblast cell line (Vero; ATCC, CCL‐81) was cultivated in Dulbecco's modification of Eagle medium (DMEM; Biosera, France), supplemented with 10% heat‐inactivated fetal bovine serum (FBS; Gibco, Thermo Fisher Scientific, MA, USA), and 1% penicillin/streptomycin (Gibco, Thermo Fisher Scientific, MA, USA). Tachyzoites of *T. gondii* were incubated with the Vero cells with a multiplicity of infection (MOI) = 1 (Nemati et al., 2022).

#### 2.3.2 Cell viability assay

To estimate the cell viability, 4 × 10^4^ of Vero cells were seeded in a 96‐well plate containing DMEM supplemented with 10% FBS, without antibiotics. Afterward, six serial concentrations of RhO‐SLNs (log^−10^ from 1 mg/mL to 100 μg/mL) were added to Vero cells. The content of negative control wells was Vero cells without treatment. After 24 h, MTT (3‐(4, 5‐dimethylthiazol‐2‐yl)‐2, 5‐diphenyltetrazolium bromide; Bio‐Idea Co, Tehran, Iran) assay was performed according to the manufacturer's instructions. In brief, 10 μL of MTT was added to the wells and incubated at 37°C for 4 h with 5% CO_2_, and the experiments were stopped by 50 μL of dimethyl sulfoxide (DMSO; Me_2_SO; 150 μL/well, Merck, Germany). Finally, the absorbance intensity was read at wavelength 570 nm using enzyme‐linked immunosorbent assay (ELISA) reader (LX800; Biotec, Winooski, VA, USA). The cell viability percentage was calculated as mentioned elsewhere (Nemati et al., 2022).

#### 2.3.3 Anti‐*Toxoplasma* activity of the RhO‐SLNs

In order to evaluate anti‐*Toxoplasma* activity, six concentrations of RhO‐SLNs were added to each experimental tube containing 2 × 10^4^ of fresh tachyzoites. After incubation at 37°C for 2 h, the viability of *T. gondii* tachyzoites was evaluated by vital staining (Khosravi et al., 2020; Mohammad Rahimi et al., 2020; Nemati et al., 2022). Tachyzoites without any treatment were considered a negative control. The intracellular anti‐*Toxoplasma* activity of RhO‐SLNs was evaluated (Nemati et al., 2022). Briefly, 1 × 10^4^ of Vero cells were seeded in a 96‐well plate containing DMEM, supplemented with 10% FBS, without antibiotics. After 24‐h incubation at 37°C and 5% CO_2_, 1 × 10^4^ of tachyzoites of *T. gondii* RH strain (MOI = 1) were added to the confluent Vero cells and incubated for an additional 24 h at 37°C and 5% CO_2_. The six concentrations of the RhO‐SLNs were added to the *T. gondii*‐infected Vero cells, and after incubation for an additional 24 h, absorbance intensity was read at wavelength 570 nm using ELISA reader (LX800; Biotec, Winooski, VA, USA). All experiences were done in duplicate. The anti‐*Toxoplasma* activity to the cell viability proportion was calculated to indicate the best concentration of the RhO‐SLNs with highest anti‐*Toxoplasma* activity and lowest cell toxicity (Mohammad Rahimi et al., 2020; Nemati et al., 2022).

GraphPad Prism software (version 8.3.0.538) was used for statistical analysis and one‐sample t‐test was employed to evaluate the statistical correlations among samples.

## 3 RESULTS

### 3.1 RhO‐SLN preparation and characterization

**FIGURE 1:**
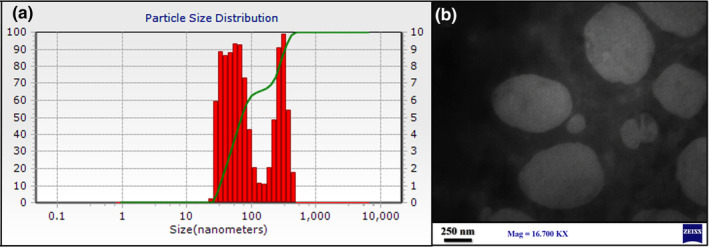
RhO‐SLN characterization based on (a) particle size analysis by DLS and (b) TEM two‐dimensional morphological description.

The average particle size and the zeta potential of RhO‐SLNs were 152.09 nm and − 15.3 mV, respectively (Figure 1a). The relatively high surface charge leads to lower particle aggregation and better size stability due to stronger electrostatic repulsion between particles. The TEM analysis of RhO‐SLNs presented smooth nanoparticles with clear edge (Figure 1b). The entrapment efficiency was 79.1%, indicating lipophilic properties of the RhO‐SLNs.

### 3.2 Release kinetics

**FIGURE 2:**
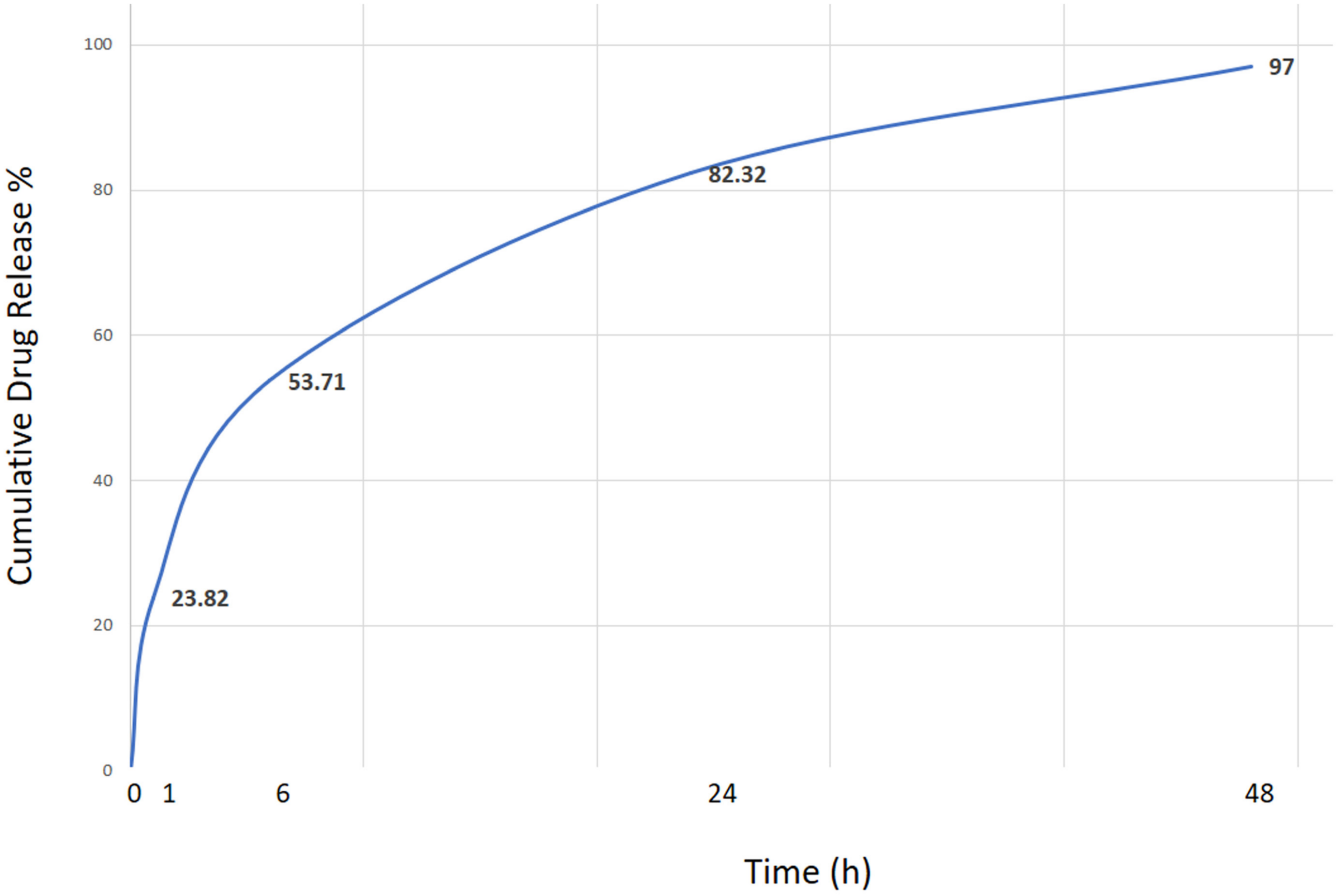
Cumulative release profile of RhO from SLNs during 48 h.

The formulation of cumulative RhO released from SLNs was performed. Maximum cumulative release reached 97% in 48 h, which reveals a relatively controlled release of RhO over time. The initial burst release at 6 h was 53.71% (Figure 2). The first‐order model showed a regression coefficient with *R* ^2^ = 0.9909 (Table 1), indicating the effect of primary concentration of RhO on the release rate, which means the release rate can be modulated with primary RhO concentrations.

**TABLE 1:** Release kinetic parameters for RhO‐SLNs based on different mathematical models.

Formulation	Zero order		First order		Higuchi		Korsmeyer–Peppas		Hixson–Crowell	
	K_0_	*R* ^2^	K_1_	*R* ^2^	K_H_	*R* ^2^	K_K_	*R* ^2^	K_Hc_	*R* ^2^
RhO‐SLN	1.75	.793	−0.03	.9909	13.8	.955	50.76	.955	0.062	.957

### 3.3 Cell toxicity of the RhO‐SLNs

**FIGURE 3:**
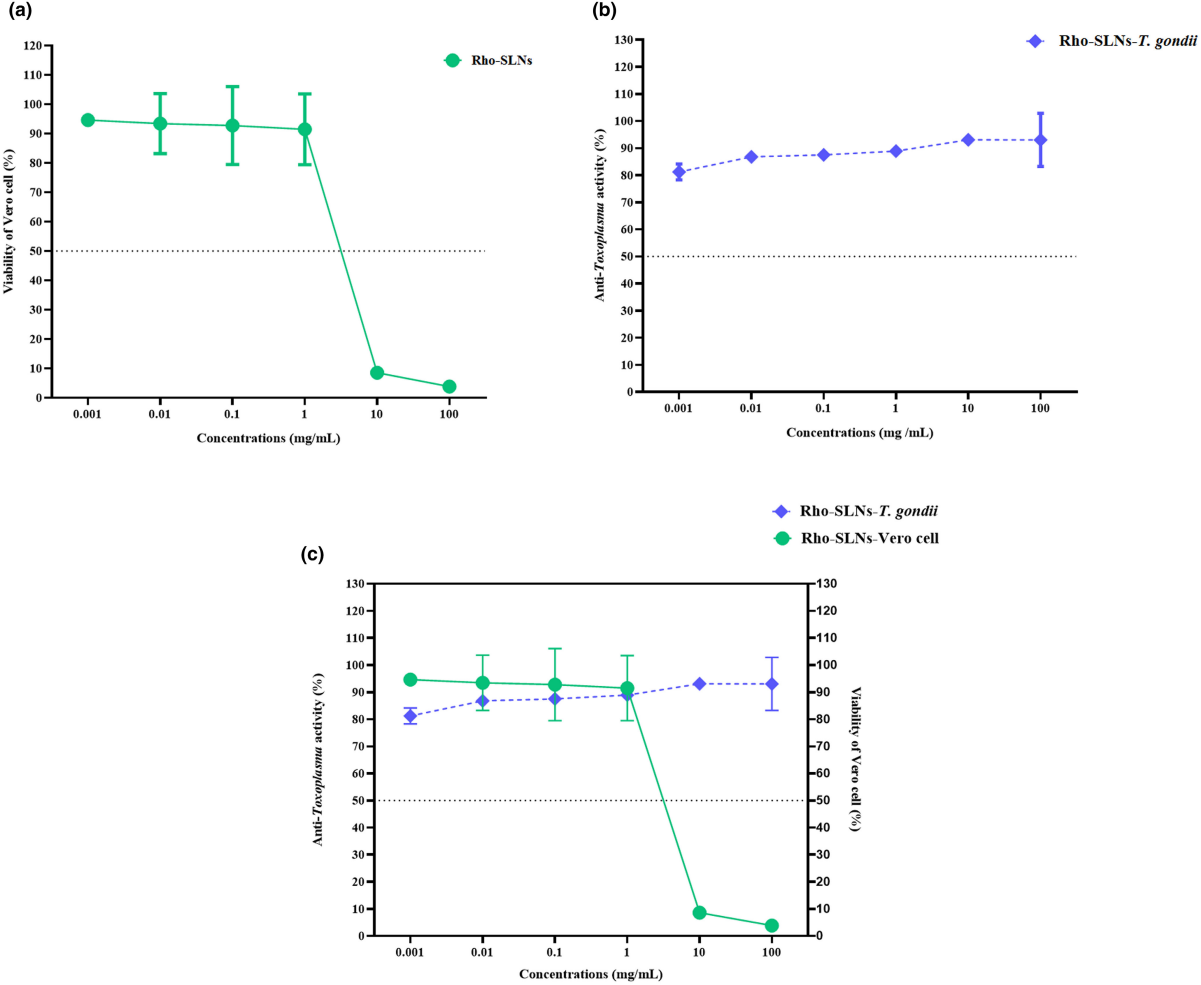
Biological assays of the RhO‐SLNs on (a) Vero cell line, which shows a statistically significant decrease in the toxicity of RhO‐SLNs, regarding the concentration (*p*‐value = .017), with a CC_50_ >10 mg/mL and (b) statistically significant anti‐*Toxoplasma* activity of RhO‐SLNs (*p*‐value <.0001) with IC_50_ >1 μg/mL. (c) The concentration of 1 mg/mL of RhO‐SLNs has high and low anti‐*Toxoplasma* activity and cell toxicity, respectively.

The cytotoxicity concentration (CC)_50_ for the RhO‐SLNs was at the concentration >10 mg/mL. Accordingly, at least 60% of Vero cells remained viable in a concentration of more than 10 mg/mL. The cell viability percentage at the lower concentration (1 μg/mL) and highest concentration (1 mg/mL) of the RhO‐SLNs ranged from 94.620 ± 1.881% (95% CI: 92.777%–96.463%) to 3.850 ± 0.622% (95% CI: 3.240%–4.460%), respectively. Changes in the cell viability were significantly concentration dependent (*p*‐value = .0173) (Figure 3a, Table 2).

**TABLE 2:** Anti‐*Toxoplasma* activity of different concentrations of RhO‐SLNs.

Concentrations mg/mL	Anti‐*Toxoplasma* activity		*p*‐value	Cell viability		*p*‐value	Ratio
	Mean ± SD (%)	95% CI (%)		Mean ± SD (%)	95% CI (%)		
100	93.050 ± 9.829	83.418–102.682	<.0001	3.850 ± 0.622	3.240–4.460	.017	24.16
10	93.100 ± 0.00	0.00		8.570 ± 2.192	6.422–10.718		10.86
1	88.900 ± 0.00	0.00		91.515 ± 12.056	79.700–103.330		0.97
0.1	87.500 ± 0.00	0.00		92.765 ± 13.301	79.731–105.799		0.94
0.01	86.800 ± 0.990	85.830–87.770		93.475 ± 10.260	83.420–103.530		0.92
0.001	81.200 ± 2.970	78.290–84.110		94.620 ± 1.881	92.777–96.463		0.85

*Note*: One‐sample *t*‐test was employed to evaluate the statistical correlations among samples.

### 3.4 Anti‐intracellular *Toxoplasma* activity of RhO‐SLNs

The results showed that less than 20% of *Toxoplasma*‐infected cells were dead in concentrations >1 μg/mL (Table 2). The viability of *T. gondii*‐infected Vero cells at the concentration of 10 and 100 mg/mL of the RH‐SLNs was 7.515 ± 6.385% (95% CI: 1.258%–13.772%) and 12.225 ± 3.769% (95% CI: 8.532%–15.918%), respectively.

### 3.5 Anti‐*Toxoplasma* activity of RhO‐SLNs and ratio analysis

The tachyzoites were checked by a microscopic examination. Accordingly, alive tachyzoites were oval [crescent] shaped with smooth surface, while dead tachyzoites were wrinkled. The RhO‐SLNs showed an anti‐*Toxoplasma* activity. Accordingly, at least 80% of *T. gondii* were dead in all concentrations with an inhibitory concentration (IC)_50_ >1 μg/mL.

The high and low inhibitory effects of RhO‐SLNs were 93.050 ± 9.83% and 81.200 ± 2.97% at 1 mg/mL and 1 μg/mL, respectively. In addition, statistical evaluations resulted in a significant concentration‐dependent anti‐ *Toxoplasma* activity of the RhO‐SLNs (*p*‐value <.0001) (Figure 3b, Table 3). The ratio analysis suggested that the concentration of 1 mg/mL with a ratio value of 0.97 had the highest anti‐*Toxoplasma* activity of 88.900 ± 0.00 (95% CI: 0.00) and lowest cell toxicity of 91.515 ± 12.056 (95% CI: 79.700–103.330), respectively (Figure 3c, Table 2).

**TABLE 3:** Anti‐intracellular *Toxoplasma* activity of different concentrations of RhO‐SLNs.

Concentrations (mg/mL)	Mean ± SD (%)	95% CI (%)	*p*‐value
100	7.515 ± 6.385	1.258–13.772	.015
10	12.225 ± 3.769	8.532–15.918	
1	87.860 ± 1.372	86.516–89.204	
0.1	97.115 ± 12.014	85.342–108.888	
0.01	104.690 ± 3.182	101.572–107.808	
0.001	106.905 ± 5.480	101.535–112.275	

## 4 DISCUSSION

In past years, herbal medicine, based on the natural plant compounds, has been developed as an alternative remedy with lower side effects than chemical agents, for a broad spectrum of communicable and noncommunicable diseases (Firenzuoli & Gori, 2007; Welz et al., 2018). Numerous studies have evaluated the effectiveness of herbal extracts or their components on infectious diseases (Chaughule & Barve, 2023; Pinn, 2001). Toxoplasmosis is a highly prevalent infectious disease, which could be fatal in subjects with immune dysfunctions such as HIV/AIDS patients and organ transplant recipients (Wang et al., 2017). Toxoplasmosis in immune‐deficient patients is associated with reactivation of latent infection, which leads to a wide variety of manifestations (Agrawal et al., 2014; Da Cunha et al., 1994). Therefore, immunocompromised patients are recommended to be treated against toxoplasmosis. Due to the side effects, resistance to current drugs, and limited efficacy, clinical treatment of toxoplasmosis and developing reliable and affordable drugs are still considerable concerns (Aspinall et al., 2002; Dunay et al., 2018; Meneceur et al., 2008). Recently, natural plant extracts and components have extremely been experienced in toxoplasmosis, which is mostly suggested as an alternative remedy with high activity, high efficiency, and low toxicity. Up to now, components and/or extracts of *Artemisia annua* (Secrieru et al., 2020), *Myristica fragrans* and *Zingiber officinale Roscoe* (Choi et al., 2013), *Dracocephalum kotschyi* (Khamesipour et al., 2021), *Azadirachta indica* (Nemati et al., 2022), *Cinnamomum camphora* (Alanazi & Almohammed, 2022), *Lippia multiflora* and *Combretum micranthum* (Benoit‐Vical et al., 2000), *Eurycoma longifolia* (Kavitha et al., 2012), *Sorghum bicolor* (Abugri et al., 2016), and *Mentha pulegium L*. and *Rubus idaeus L* (Mohammad Rahimi et al., 2020) have been reported to be effective on toxoplasmosis.

*Rosa rubiginosa*, known as rose hips, are widely used in cosmetic products and the food industry (Franco, Pinelo, et al., 2007; Loetscher et al., 2013; Santana et al., 2016). Rose hip fruits are particularly rich in antioxidant compounds, minerals, vitamin C, ascorbic acid, carotenoids, and phenols (Demir et al., 2014; Ercisli, 2007; Franco, Pinelo, et al., 2007; Franco, Sineiro, et al., 2007; Roman et al., 2013). The effects of rose hips extract on wound healing in rats were evaluated (Lei et al., 2019). Moreover, antimicrobial effects of ethanolic and methanolic extracts of RhO have been evaluated and the results were promising (Olech et al., 2017; Talib & Mahasneh, 2010; Vlaicu et al., 2017; Yi et al., 2007). For example, Rovnã et al. (2015) investigated the antibacterial effects of ethanolic and methanolic extracts of RhO on *Escherichia coli*, *Pseudomonas aeruginosa*, *Aspergillus niger, Fusarium culmorum*, and *Alternaria alternata*, and the results showed high effectiveness of the ethanolic extract on all evaluated microorganisms.

A number of studies have investigated the anti‐*Toxoplasma* activity of plant extracts and products, which have presented the ability of these natural compounds to inhibit the proliferation of *Toxoplasma* in vitro and in vivo (Khamesipour et al., 2022; Nemati et al., 2022; Rosenberg et al., 2019). However, the effects of the rose hip extract on *T. gondii* are not clear. Many nanotechnology‐based formulations have been reported to improve the safety and efficacy of drugs in treating toxoplasmosis in vitro and in vivo (Assolini et al., 2017; Leyke et al., 2012). Teimouri et al. (2018) investigated the potential of various molecular weights and concentrations of chitosan nanoparticles (CS NPs), against tachyzoites of *T. gondii* RH strain. Their results showed that CS NPs were properly formulated and can be used as an alternative natural medicine in the treatment of toxoplasmosis.

During the last decades, nanotechnology has been widely developed in drug delivery systems. Nanoparticles with high surface‐to‐volume ratio provide higher surface area and dissolution rate for particles (Barbosa et al., 2019). In addition, employing suitable nanocarriers overcomes the solubility and bioavailability limitations of conventional drugs (Alshawwa et al., 2022; Arshad et al., 2021; Din et al., 2017). Nanocarriers increase the solubility of the hydrophobic agents and improve target delivery of drugs (Jafarpour Azami et al., 2021). Polymeric nanoparticles, liposomes, dendrimers polymeric micelles, carbon nanotubes, and SLNs are well‐known nanocarriers, of which SLNs are ideal candidates for developing enclosed carriers for different types of therapeutic agents (Bayón‐Cordero et al., 2019). SLNs, as a proper option for medicinal purposes, present multiple advantages such as low toxicity, high stability, high biocompatibility and biodegradability, high release kinetics, and proper pharmacokinetics and pharmacodynamics (Nemati et al., 2024). In addition, SLNs are able to control the drug release (Scioli Montoto et al., 2020; Thorn et al., 2021). Due to their biopharmaceutical aspects, SLNs possess great advantages in certain specific therapeutic fields like cancer therapies (Scioli Montoto et al., 2020). da Rocha et al. (2020) investigated the antitumor effects of SLNs‐docetaxel (SLN‐DTX) to prevent tumor growth of 4 T1 murine mammary carcinoma cells. They showed that SLN‐DTX not only exhibited high antitumor effects, but also reduced the tumor size compared to control, and prevented spontaneous lung metastasis test mice. From a toxicological point of view, Nassimi et al. (2009) showed high biosafety of SLNs for human lung as a desirable drug delivery system (DDS), based on the human alveolar epithelial cell line (A549) and murine precision‐cut lung slices (PCLS). Furthermore, Khosravi et al. (2020) synthesized mannosylated paromomycin‐loaded SLNs (PM‐SLN‐M) for evaluating anti‐*Toxoplasma* effects. As a result, PM‐SLN‐M showed high anti‐*Toxoplasma* activity and low cytotoxic effects on Vero cells compared to control groups.

Nevertheless, encapsulation of plant extracts in SLNs, as a robust nanocarrier, has already been employed to overcome the limitations due to the multidrug resistance and to improve target delivery of antibiotics (Christaki et al., 2020; González‐Paredes et al., 2019). Few studies have combined herbal extracts with SLNs for the treatment of parasites. Nemati et al. (2022) fabricated neem oil‐SLNs (NeO‐SLNs) and showed high ability of the nanodrug in killing of *T. gondii* tachyzoites in a concentration of 100 μg/mL with cell toxicity lower than 20%. According to our findings, RhO‐SLNs in a concentration of 1 mg/mL showed higher anti‐*Toxoplasma* activity with cell toxicity lower than 10%. Comparing the findings of ratio analysis with our previously published study suggests that the best concentration for RhO‐SLNs was 1 mg/mL, while for NeO‐SLNs, it was 100 μg/mL. This finding suggests that although the anti‐*T. gondii* effects are lower, the cell biosafety of RhO‐SLNs is higher than NeO‐SLNs. In addition, the EE% of RhO‐SLNs was 79.1%, while it was 71.6% for NeO‐SLNs (Nemati et al., 2022).

## 5 CONCLUSION

Our findings suggest the therapeutic aspects of RhO‐SLNs. RhO‐SLNs exhibited potent anti‐*Toxoplasma* activity while its cell toxicity on Vero cells was acceptable. This study is a primary experiment, which suggested that encapsulation by a nano‐vehicle such as SLNs may increase antimicrobial activity and decrease cell toxicity of herbal extracts.

## AUTHOR CONTRIBUTIONS

**Hanieh Mohammad Rahimi:** Methodology (equal); software (equal); visualization (equal). **Zahra Hesari:** Investigation (equal); methodology (equal); validation (equal); visualization (equal). **Elnaz Sadat Mirsamadi:** Methodology (equal); resources (equal). **Sara Nemati:** Conceptualization (equal); visualization (equal); writing – original draft (equal). **Hamed Mirjalali:** Conceptualization (equal); supervision (equal); writing – review and editing (equal).

## FUNDING INFORMATION

This study was financially supported by Shahid Beheshti University of Medical Sciences, Tehran, Iran (Grant no: 43007082).

## CONFLICT OF INTEREST STATEMENT

The authors declare that they have no conflict of interest.

## ETHICS STATEMENT AND CONSENT TO PARTICIPATE

The study was performed in accordance with the relevant guidelines and declaration. The study was approved by the Ethics Committee of Shahid Beheshti University of Medical Sciences, Tehran, Iran (IR.SBMU.RIGLD.REC.1402.007).

## CONSENT FOR PUBLICATION

Informed consent was obtained from all subjects and/or their legal guardian(s). All authors declare that they have seen and approved the submitted version of this manuscript.

## Data Availability

All generated data from the current study are included in the article.
